# Molecular Determinants Underlying the Anti-Cancer Efficacy of CD38 Monoclonal Antibodies in Hematological Malignancies

**DOI:** 10.3390/biom12091261

**Published:** 2022-09-08

**Authors:** Nurulhuda Mustafa, Muhamad Irfan Azaman, Giselle G. K. Ng, Wee Joo Chng

**Affiliations:** 1 Department of Medicine, Yong Loo Lin School of Medicine, National University of Singapore, Singapore 117597, Singapore; 2 Cancer Science Institute of Singapore, National University of Singapore, Singapore 117599, Singapore; 3 Programme in Emerging Infectious, Duke-NUS Medical School, Singapore 169857, Singapore; 4 Department of Hematology-Oncology, National University Cancer Institute, Singapore 119074, Singapore

**Keywords:** CD38, immunotherapy, blood malignancies, daratumumab, drug combination, extracellular vesicles, miRNA

## Abstract

CD38 was first discovered as a T-cell antigen and has since been found ubiquitously expressed in various hematopoietic cells, including plasma cells, NK cells, B cells, and granulocytes. More importantly, CD38 expression levels on malignant hematopoietic cells are significantly higher than counterpart healthy cells, thus presenting itself as a promising therapeutic target. In fact, for many aggressive hematological cancers, including CLL, DLBCL, T-ALL, and NKTL, CD38 expression is significantly associated with poorer prognosis and a hyperproliferative or metastatic phenotype. Studies have shown that, beyond being a biomarker, CD38 functionally mediates dysregulated survival, adhesion, and migration signaling pathways, as well as promotes an immunosuppressive microenvironment conducive for tumors to thrive. Thus, targeting CD38 is a rational approach to overcoming these malignancies. However, clinical trials have surprisingly shown that daratumumab monotherapy has not been very effective in these other blood malignancies. Furthermore, extensive use of daratumumab in MM is giving rise to a subset of patients now refractory to daratumumab treatment. Thus, it is important to consider factors modulating the determinants of response to CD38 targeting across different blood malignancies, encompassing both the transcriptional and post-transcriptional levels so that we can diversify the strategy to enhance daratumumab therapeutic efficacy, which can ultimately improve patient outcomes.

## 1. Introduction 

Antibody-based immunotherapy has emerged amongst the most successful treatment strategies in cancer and is now incorporated as a mainstay treatment option in conjunction with chemotherapy, surgery, and radiation [1]. The monoclonal antibody has high target affinity and specificity, which enables selective disruption of pro-tumoral signaling pathways while simultaneously recruiting the immune system to generate long-term antitumor responses, thus favoring superior and durable treatment outcomes with manageable side effects for patients [2]. CD38-targeting monoclonal antibodies against cancer exemplify one such success story in the clinic. 

Daratumumab is a first-in-class, IgG1κ human CD38-targeting monoclonal antibody first approved by FDA for the treatment of multiple myeloma (MM) in 2015. It has since been incorporated into the treatment regimen for both relapsed/refractory and newly diagnosed multiple myeloma patients, so far demonstrating outstanding improvements in clinical efficacy, both as a single agent and in combination with other chemotherapeutics [3,4,5,6]. Isatuximab is a novel CD38-targeting antibody raised against a different epitope from daratumumab and is differentiated by the direct cytotoxic effect it can induce in tumors, as well as stronger inhibition of the tumor suppressive NADase role of CD38 in vitro [7,8]. Isatixumab has currently been approved in combination with carfilzomib and dexamethasone, or pomalidomide and dexamethasone for the treatment of adult patients with relapsed or refractory MM who have received one to three prior lines of therapy [9,10]. Other CD38-targeting antibodies MOR202 [11] and TAK079 [12] are also currently under preclinical and clinical evaluation for potential incorporation in the clinic. 

Enhanced expression of the CD38 membrane protein has also been reported in other aggressive hematological malignancies, including chronic lymphocytic leukemia (CLL), T-cell acute lymphoblastic leukemia (T-ALL), diffuse large B-cell lymphomas (DLBCL), and T and NK lymphomas (NKTL) [13,14,15,16,17]. This elevated CD38 expression has been associated with poorer prognosis and a more aggressive cancer phenotype, which is an outcome of the cancer cell hijacking underlying CD38-driven functions to create both a pro-tumoral extracellular environment and a cancer-permissive intracellular milieu [18,19,20]. This has generated an impetus for the evaluation of CD38-targeting therapeutics in such cancers. However, early-phase clinical trials have reported mostly modest efficacy with daratumumab treatment, suggesting that more work needs to be carried out to understand the determinants of response to CD38 targeting [21,22]. In addition, long-term and widespread treatment with daratumumab has led to resistance in a subset of MM patients and efforts are underway to identify strategies that can enhance daratumumab efficacy. 

This review will describe the pattern of CD38 expression across different hematological malignancies and study unique molecular factors regulating CD38 expression and functions. This will highlight potential strategies that can be utilized in combination with CD38-targeting antibodies to enhance therapeutic responses in CD38-driven hematologic malignancies. 

## 2. CD38 Protein Expression and Function in Healthy Cells 

CD38 is ubiquitously expressed in multiple human tissues, with the highest expression detected in hematopoietic tissues, such as bone marrow and lymph nodes. Originally identified as an antigen on the surface of T cells [23], CD38 expression has been reported in a wide range of hematopoietic cells, including B cells, NK cells, macrophages, granulocytes, neutrophils, and platelets, as well as nonhematopoietic cells, including the prostate epithelium, pancreatic beta cells, and in organs such as the brain, the liver, and the kidney [24,25,26,27]. 

**Figure 1:**
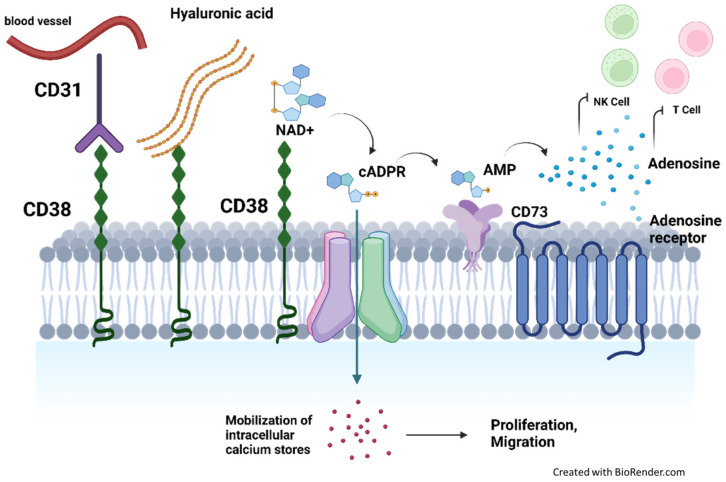
Functional roles of CD38. CD38 can mediate adhesion through binding with (1) hyaluronic acid in the extracellular matrix or through (2) binding with its cognate ligand CD31 to mediate adhesion and transendothelial migration. (3) Importantly, the ecto-enzymatic domain of CD38 catabolizes NAD+ into cADPR, which can enter the cell to mobilize calcium stores and modulate numerous cell signaling pathways. cADPR can also be hydrolyzed to ADPR, and then subsequently adenosine upon colocalization with CD73/203a. Adenosine is bound to purinergic receptors to suppress NK and T-cell activation.

Structurally, CD38 is a transmembrane glycoprotein with a large bifunctional extracellular catalytic domain and single transmembrane segment near its N-terminus [24]. This structural conformation is well adapted for the diverse and pleiotropic functions that CD38 mediates in regulating cell adhesion, migration, proliferation, intracellular signal transduction, metabolic reprograming, modulation of the cell microenvironment through inflammation, and immunosuppression (Figure 1).

CD38 knockout mice models convey an overall insight as to the key functions of this protein. While CD38 null mice appear healthy, fertile and unburdened by gross defects, they, however, demonstrate a dramatic reduction in NAD+ glycohydrolase activity in the spleen and a complete abolishment in the liver and brain. Interestingly, this loss of NAD+ glycohydrolase activity did not significantly impact the development, maintenance, or establishment of population ratios of hematopoietic stem cells. Notably though, CD38 null mice exhibited marked deficiencies in the antibody responses to T-cell-dependent protein antigens, thus suggesting that CD38 may play a role in regulating humoral immune responses [6,28]. 

In human CD38 studies, its function as an adhesion molecule was first established by receptor binding to hyaluronic acid in the extracellular matrix through a hyaluronate binding motif [29]. Subsequently, it was demonstrated that CD38 binds to a cognate ligand CD31 and mediates adhesion and transmigration between leukocytes and the endothelial wall, thereby promoting the activation and proliferation of these cells [30]. CD38 also regulates the migration of dendritic cell (DC) precursors from blood to peripheral sites, as well as the migration of mature DCs to lymph nodes in response to CCL2, CCL19, CCL21, and CXCL12 chemokines [31]. 

The extracellular enzymatic (ecto-enzymatic) domain of CD38 can mediate two independent reactions, one as an NAD glycohydrolase and also as a ADP ribosyl cyclase, eventually catabolizing NAD+ into two different products which are essential for intracellular calcium homeostasis. It catalyzes the formation of adenosine diphosphate ribose (ADPR), cyclic ADPR, and nicotinamide from NAD+ under neutral pH or nicotinic acid adenine dinucleotide phosphate (NAADP+) under acidic conditions [32,33,34,35]. cADPR and NAADP are structurally and functionally distinct messengers that mobilize calcium from endoplasmic reticulum and lysosomal calcium stores, respectively, leading to modulation of intracellular signaling pathways [36,37]. This ecto-enzymatic function also links to an immune-suppressive role for CD38 through the regulation of extracellular adenosine, which is a byproduct from the catabolism of NAD+ and ATP [38]. Adenosine has been implicated in the control of inflammation and immune response through purinergic receptor binding, which may be exploited by malignant cells for immune escape [39]. 

## 3. CD38-Mediated Tumor-Promoting Mechanisms and Expression in Hematological Cancers 

Anomalous CD38 expression in hematologic malignancies correlates with hyper-proliferation of cells, dysregulation of intra- and extracellular metabolic pathways, formation of a tumor permissive microenvironment, and disease progression, thus making CD38 an attractive target for antibody-based therapeutics [16,18,19,20,40]. Here, we examine how CD38-mediated mechanisms have been hijacked for tumorigenesis in the various hematological cancers and how these can be potentially exploited to enhance monoclonal antibody-mediated antitumor responses. The summary of these mechanisms can be found in Table 1. 

### 3.1. CD38 in Multiple Myeloma

**Figure 2:**
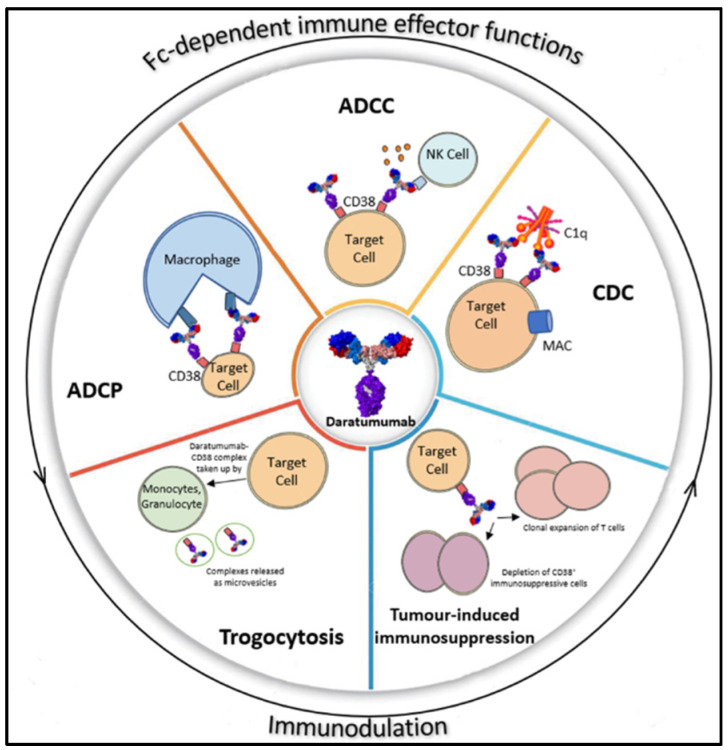
A broad spectrum of mechanisms of action of daratumumab. Daratumumab triggers Fc-dependent immune effector mechanisms that comprise of CDC, ADCC, and ADCP. The Fc tail of daratumumab with the Fc gamma receptors (FcγRs) present on immune effector cells leads to activation of these immune cells and subsequent lytic killing of MM cells. Lysis and depletion of CD38+ immune suppressor cells, such as Tregs, also occur via the same process, leading to immunomodulation of the tumor niche and clonal expansion of cytotoxic T cell. CD38–daratumumab complexes that are formed are transferred from MM cells to monocytes and granulocyte in a process known as trogocytosis, thereby modulating CD38 expression on immune cells.

Multiple myeloma is the malignant growth of clonal plasma cells primarily located in the bone marrow and, also, the second most common hematological malignancy. Malignant plasma cells from any stage of this disease express uniformly high levels of the CD38 antigen, higher than healthy myeloid and lymphoid cells (2). This differentiated expression provides the basis for the good clinical outcomes that have been achieved with daratumumab treatment in patients. Through Fc-receptor-dependent mechanisms, daratumumab recruits the immune system and eliminates CD38-expressing tumor cells via a broad spectrum of mechanisms (Figure 2), including complement-dependent cytotoxicity (CDC) [41], antibody-dependent cellular cytotoxicity (ADCC), antibody-dependent cellular phagocytosis (ADCP), induction of apoptosis [42,43], and an indirect immune modulatory effect moderated by the depletion of suppressive CD38+ regulatory T cells [44]. In contrast to MM, the clonal plasma cell in amyloidosis (AL) has a lower proliferation index [45]. Despite this, AL clonal plasma cells express surface CD38 [46], allowing monoclonal antibodies, such as daratumumab, to inhibit light-chain production [47,48]. A considerable amount of malignant clone of plasma cells in Waldenström macroglobulinemia patients expresses CD38 (40–70%) [49,50,51], explaining the rationale for a recently completed clinical trial to treat Waldenström macroglobulinemia patients with daratumumab [52].

While the anticancer efficacy of daratumumab treatment can be attributed to the abovementioned immune-mediated mechanisms, additional studies have highlighted that the depth and durability of the antitumor daratumumab response was also due to the disruption of onco-promoting signals mediated by CD38 [40,43,44,53,54,55,56].

#### 3.1.1. CD38 Increases Capacity for Oxidative Phosphorylation 

Compelling evidence has been provided for the role of CD38 in directly promoting MM survival through mechanisms supporting bioenergetic plasticity of the myeloma cell. Marlein et al. demonstrated that the CD38 mediates mitochondrial transfer between bone marrow stromal cells (BMSC) and myeloma cells through tumor-derived tunneling nanotubes, thus promoting MM proliferation by augmenting mitochondrial-based oxidative phosphorylation [54]. CD38-directed transfer of mitochondria has also been observed between astrocytes and damaged neurons after a stroke [53].

#### 3.1.2. CD38 Mediates Immunosuppression by Producing Elevated Levels of Adenosine

In the bone marrow niche, MM cells undergo metabolic reprograming, which upregulates NAD+, lactate, and H+ levels [40]. Through the ecto-enzymatic activity of CD38, NAD+ is converted to cyclic ADPR and then ADPR, which is a substrate for adenosine formation. Bone marrow plasma aspirates contain elevated levels of adenosine, which increase as the disease progresses [20]. Adenosine leads to tumor growth and skews the immune cells towards an immunosuppressive phenotype. It binds purinergic cell receptors that transduce autocrine and paracrine signals, which can inhibit effector T cells, NK cells, and dendritic cells and conversely stimulate the upregulation of regulatory T cells and myeloid-derived stromal cells so as to hinder an immune-mediated response against the tumor [55,56].

Using a recombinant CD38 protein, van de Donk et al. showed that the anti-CD38 monoclonal antibody Dara can reverse the tumor-promoting activities of the CD38 ectoenzyme by reducing ADPR cyclase activity and inducing cADPR hydrolase activity, thus increasing NAD+ and ADPR levels and decreasing cADPR levels [43].

#### 3.1.3. CD38 Expression on Immune Suppressor Cells Promote a Pro-Tumoral MM Niche

Studies in patients with multiple myeloma have found that CD38+ T regulatory (Treg) cells are more suppressive than CD38− Treg cells, consisting of a novel subpopulation of Tregs (CD41CD251CD127dim), which demonstrated superior autologous T-cell inhibitory capacities [44,57]. Isatuximab was able to preferentially suppress proliferation and cytokine production in Tregs and restore proliferation and function of T effector cells. It also augments MM cell lysis by CD8+ T and natural killer cells [58]. 

CD38 is also expressed on a large subset of other immune suppressor cells, such as regulatory B cells and myeloid-derived suppressor cells. The presence of these cells in the tumor microenvironment contribute to tumor growth, immune evasion, angiogenesis, metastasis, and production of suppressive cytokines, which can be reversed upon addition of daratumumab. Daratumumab binding to CD38 on the immune-suppressive cells leads to the depletion of these populations and subsequent expansion of CD4+ T helper cells, CD8+ cytotoxic T cells, and increases T cell clonality [44]. Patients also showed increased T-cell responses against pre-existing viral and alloantigens.

### 3.2. CD38 in Chronic Lymphocytic Leukemia

Chronic lymphocytic leukemia (CLL) is a highly heterogenous disease, which is characterized by a dynamic balance between the atypical accumulation of indolent lymphocytes in the periphery and proliferation stimuli in the bone marrow, which determines disease course and aggressiveness. The most favorable conditions for expansion of CLL clones exist in discrete anatomic sites, such as proliferation centers in the lymph node and bone marrow, where leukemic cells come into contact with accessory cells and the suitable array of cytokines and chemokines. Identification of markers that may correlate with more aggressive CLL cell subsets is, therefore, crucial and, in this context, increase in CD38 expression through the course of the disease has been regarded as a potential marker for the transition to a more aggressive phenotype [59,60,61]. CD38 is expressed by approximately 27–46% of patients [62,63]. In CLL, CD38 expression is a negative prognostic indicator associated with inferior CLL outcome [62,63]. Patients who are CD38-positive were characterized with an unfavorable clinical course with a more advanced disease stage, poor responsiveness to chemotherapy, and shorter survival. Patients with CD38(+) samples have significantly aggressive disease regardless of their clinical stage, in contrast to the CD38-negative group who required minimal treatment and had prolonged survival.

#### 3.2.1. CD38 Promotes Migration towards Proliferative Niches through Adhesion and Cytokine Production

In vivo, CD38 appears to act as a molecular compass that directs leukemic cells to specialized niches based on observations that the number of CD38 molecules expressed in BM and LN is higher than in circulating lymphocytes. Homing of chronic lymphocytic leukemia (CLL) cells to sites favoring growth, a critical step in disease progression, is principally coordinated by the CXCL12/CXCR4 axis. Through potential colocalization with CXCR4 on the membrane, CD38-positive CLL cells demonstrate heightened chemotaxis to CXCL12 signaling [19]. 

A direct role for CD38 in enhancing CD49d-mediated adhesion processes in CLL has also been elucidated. The proposed model for a prosurvival circuitry operating in CD38+CD49d+ CLL revolves around CD38/CD31 inducing the release of CCL3 and CCL4 by CLL cells. These chemokines attract CD68+ myeloid cells expressing the CCL3 receptor, which then release TNFα and other cytokines, thereby increasing interactions with VCAM-1/CD49d, which promotes survival of CD49d-expressing CLL cells [64,65]. Additionally, CD38 physically colocalizes with CD49d to Vav-1, which potentiates its phosphorylation and, thus, the activation of the integrin signaling pathway, which facilitates homing. Lastly, Mele, et al. recently demonstrated that CD38-expressing CLL cells exhibit a calcium-mediated Rap1 GTPase activation [66], which is known to have a crucial role in leukocyte trafficking and homing.

#### 3.2.2. CD38 Directly Stimulates Growth and Survival Signals

CD38 has also been shown to colocalize with CD19 and CD81 in membrane lipid raft, which facilitates the transduction of BCR signals [67]. Gene expression profiling of CD38+ and CD38− members of the same clone highlighted elevated levels of VEGF and Mcl-1 in CD38+ cells, conferring a survival advantage potentially from CD38-mediated interactions with the tumor microenvironment [65]. 

#### 3.2.3. CD38+ CLL Cells Exhibit Intrinsic Molecular Characteristics in Favor of Promoting Oncogenesis

Other characteristics of the CD38+ and CD38− CLL subgroups are variable telomere lengths, telomerase level, and expression of COX-2 changes over time [68,69]. CD38/CD31 interaction results in a genetic signature, with proliferation and migration emerging as the main elements characterizing this receptor/ligand system. In vitro activation through CD38 drives CLL proliferation and chemotaxis via a signaling pathway that includes ZAP-70 and ERK1/2. Finally, CD38 is under a polymorphic transcriptional control after external signals. Consequently, CD38 appears to be a global molecular bridge to the environment, promoting survival/proliferation over apoptosis.

#### 3.2.4. CD38 Expression on Immune Suppressor Cells Promote a Pro-Tumoral CLL Niche

Patients with CLL exhibit profound immunosuppression, which can be attributed to the presence of a large population of Treg cells and Breg, such as CLL cells. High CD38 expression on these cells results in ablation of cells, with CD38-targeted therapeutics resulting in repopulation of the immune milieu with immune-reactive cells which promote an antitumor response [62].

### 3.3. CD38 in Aggressive Non-Hodgkin Lymphomas

Mantle cell lymphoma (MCL) is a rare type of aggressive non-Hodgkin lymphoma (NHL) [70]. Despite an intensive treatment based on strong chemotherapy regimens and autologous stem cell transplantation, virtually all patients eventually relapse [71]. CD38 expression is found on 90% of the cases of MCL and correlates with nodal involvement and poorer prognosis [72]. Additionally, CD38 expression appears to mediate some resistance to Bortezomib [73]. Altogether, in MCL, high CD38 expression promotes clonal B-cell accumulation and may, therefore, represent an attractive therapeutic target in this disease. Diffused large B cell lymphoma (DLBCL) is the most common NHL. Approximately 40% of patients do not respond to first-line therapy and develop a refractory course [74,75]. CD38 expression is abnormally high in DLBCL and can be detected in 80% of the cases [22]. Expression of CD38 on the surface appears to correlate with aggressiveness of the tumor and, in de novo DLBCL, high CD38 expression is associated with significantly worse progression-free survival and poor overall survival [13,76]. There have been no oncogenic mechanisms directly attributed to CD38 in DLBCL so far; however, its expression on aggressive variants of DLBCL suggests that it may support a proliferative phenotype. CD38 appears to be robustly detected in the lymphocytic infiltrate from the tumor microenvironment of classical Hodgkin lymphoma, reminiscent of powerful immunosuppressive roles it mediates in the bone marrow niche of MM [77].

### 3.4. CD38 in T and NK Lymphomas

T and natural killer (NK) lymphomas are a group of lymphoid tumors that are highly malignant and generally associated with poorer outcomes. In peripheral T-cell lymphomas and extranodal NK/T-cell lymphoma (ENKTL), prognosis remains relatively dismal, with a 5-year overall survival rate of approximately 30 to 50% [78]. Variable levels of CD38 expression have been detected in approximately 80% of angioimmunoblastic T-cell lymphoma (AITL) and 60% of peripheral T-cell lymphoma, not otherwise (PTCL-NOS), thus providing a rationale for novel treatment with anti-CD38 antibodies [79]. CD38 is also expressed by the majority of ENKTL nasal type, a rare and aggressive subtype of mature T- and NK lymphomas commonly associated with maturity and Epstein Barr virus (EBV) infection [14,80]. CD38 expression was shown to associate with poorer prognosis [80]. Indeed, subsequent reports have demonstrated that, for the subset of patients which express high levels of CD38, daratumumab treatment is able to inhibit NKTL survival and growth in both the cell lines and in a cancer xenograft model [14]. It clearly highlights that the ratio of CD38: complement inhibitory protein is an important predictor of susceptibility to daratumumab treatment and not absolute CD38 levels alone. Phase 2 clinical trials of daratumumab monotherapy in relapsed/refractory NKTL demonstrated that it was well tolerated and achieved an overall response rate of 25.0% [21].

**Table 1:** Schematic overview of oncogenic mechanisms mediated by CD38 in different blood malignancies.

CD38 Function	How This Is Hijacked to Promote Cancer	Type of Blood Malignancy
**Ecto-enzymatic NADase activity**	Elevated levels of adenosine suppress activity of effector immune cells and stimulate activity of regulatory T cells and myeloid-derived suppressor cells	MM, DLBCL, T-ALL
	Increased enzymatic activity increases production of cADPR and NAADP calcium messengers, which promote survival, trafficking, and homing	MM, CLL, AML
**Adhesion**	Formation of nanotubes to mediate mitochondrial transfer from BMSC to promote oxidative phosphorylation	MM
**Cell surface receptor/antigen**	Increased expression on immune suppressor cells, which intensifies cell suppressive phenotype and promotes formation of immune-suppressive tumor niches	MM, CLL
	Chemokine-mediated migration towards proliferative niches	CLL, AML
	Colocalization with other receptors to directly transduce survival signaling	CLL
	Biomarker for poor prognosis	CLL, MCL, DLBCL, PTCL, NKTL

MM, multiple myeloma; DLBCL, diffuse large B-cell lymphoma; T-ALL, T-cell acute lymphoblastic leukemia; CLL, chronic lymphocytic leukemia; AML, acute myeloid leukemia; MCL, mantle cell lymphoma; PTCL, peripheral T-cell lymphoma; NKTL, natural killer T-cell lymphoma.

### 3.5. CD38 in Acute Myeloid Leukemia (AML) and T cell Acute Lymphoblastic Leukemia (T-ALL)

AML is the most common form of acute leukemia in adults. CD38 expression assessed on 37 AML and 12 T-ALL patients highlighted that CD38 expression is heterogenous in AML, more uniform in T-ALL, and that expression did not correlate with progressive disease [81]. Another in-depth analysis of CD38 expression in a large cohort of T-ALL at diagnosis during chemotherapy and at relapse found that CD38 expression was positive in 97.9% of diagnosed patients, 88.7% patients with minimal residual disease (MRD) and 82.9% relapsed samples [82]. No significant difference was noted in CD38 expression between pediatric versus adult or between diagnostic versus MRD and diagnostic versus relapsed paired samples. CD38 is robustly expressed in T-ALL blasts despite exposure to cytotoxic chemotherapy, making it a potentially effective target for anti-CD38-monoclonal therapy. The role of CD38 in these diseases, however, is still not fully understood. Preclinical studies have highlighted the potential efficacy for daratumumab in these diseases [15,83]. Findings from one of the studies propose that, mechanistically, daratumumab efficacy can be attributed to ADCP and the disruption of CD38-mediated trafficking and migration of AML. Daratumumab treatment in an AML xenograft model impaired the homing of AML cells to the bone marrow and spleen by three- to fivefold. It was also able to reduce the capacity of AML transendothelial migration by 50% [83]. In a more recent study, combining daratumumab with a CD47-blocking antibody substantially prolonged survival as compared to single treatments, and it was hypothesized that the CD47 blockade was able to overcome the immunosuppressive effects on ADCP mediated by dysregulated CD38 expression in T-ALL [84].

### 3.6. Clinical Studies of CD38-Targeting Antibodies in Hematological Malignancies (Table 2)

CD38-targeting antibodies are transforming the treatment landscape, particularly for multiple myeloma patients, having demonstrated profound anticancer efficacy coupled with a relatively manageable toxicity profile. Currently, two anti-CD38 monoclonal antibodies, daratumumab and isatuximab, are approved for MM in the clinic [85,86,87,88], whilst a third, MOR 202, is presently being evaluated in clinical trials [11]. Studies show that daratumumab stimulates stronger induction of Fc-dependent immune effector mechanisms CDC and ADCP as compared to isatuximab; however, isatuximab is unique in its ability to directly induce apoptosis in the cancer cells and inhibit tumor-promoting enzyme-mediated activities of CD38 due to direct binding to its ecto-enzymatic site [89].

Substantial clinical improvement has been observed, especially with the combination of current myeloma therapeutic regimens and CD38-targeting antibodies. The addition of either daratumumab or isatuximab to the backbone of immunomodulatory drugs (lenalidomide and pomalidomide) plus dexamethasone significantly improved progression-free survival in phase 3 POLLUX, APOLLO, and ICARIA-MM clinical trials, respectively [9,86,87]. Marked patient responses were also observed, including in patients 65 years and older, when daratumumab or isatuximab was added to the backbone cocktail of proteasome inhibitor (bortezomib and carfilzomib) and dexamethasone in CASTOR, CANDOR, and IKEMA clinical trials [5,10,90,91]. 

In other hematological malignancies, the clinical evaluation of CD38-targeting antibodies is still in its early stages. Treatment with daratumumab as a single agent in relapsed refractory NKTL patients confirmed no new safety concerns and 8 out of 32 patients demonstrated a partial response [21]. However, none of the responders achieved complete response and duration of response was short, with a median of 55 days. Currently, there is an active phase 2 trial studying the combination of isatuximab and cemiplimab (PD1 inhibitor) in this malignancy (NCT04763616 (ICING)). Blocking PD-L1, which is expressed on the tumor cells of ENKTL and ANKL patients, can restore immune effector function in the tumor microenvironment and may produce a synergistic effect through the augmentation of the immune-mediated anticancer response triggered by Isatixumab. 

Daratumumab monotherapy in the three-arm CARINA trial against relapsed/refractory mantle cell lymphoma, diffuse large B -cell lymphoma, and follicular lymphoma was terminated at Stage 1 of the study [22]. This was due to the failure to meet futility thresholds, which were defined by overall response rate (ORR) of 50% and 30% in FL and DLBCL, respectively. Similarly, the phase 2 study of isatuximab monotherapy in refractory T-acute lymphoblastic leukemia (T-ALL) or T-lymphoblastic lymphoma (T-LBL) displayed low efficacy as a single agent and was subsequently terminated [92].

**Table 2:** Key clinical studies of CD38-targeting antibodies in hematological malignancies.

Tumor Type	Study Title	Phase	Drug Regimen	Median PFS	Ref
MM	NCT02076009, POLLUX	3	Dara-Len-Dex vs. Len-Dex	44.5 vs. 17.5 months	[85,86]
	NCT03180736, APOLLO	3	Dara-Pom-Dex vs. Pom-Dex	12.4 vs. 6.9 months	[87]
	NCT02136134, CASTOR	3	Dara-Bort-Dex vs. Bort-Dex	60.7 vs. 26.9 months	[5,93]
	NCT03158688, CANDOR	3	Dara-Carfil-Dex vs. Carfil-Dex	28.6 vs. 15.2 months	[91]
	NCT01749969	1b	Isa-Len-Dex	8.5 months	[88]
	NCT02990338, ICARIA-MM	3	Isa-Pom-Dex vs. Pom-Dex	11.5 vs. 6.5 months	[9]
	NCT03275285, IKEMA	3	Isa-Carfilz-Dex vs. Carfilz-Dex	35.7 vs. 19.2 months	[10]
	NCT01421186	1b/2a	MOR202-Len-Dex	not reached after 24 months	[11]
	NCT01421186	1b/2a	MOR202-Pom-Dex vs. Mor Dex	17.5 vs. 8.4 months	[11]
NKTL	NCT02927925	2	Dara single agent	55 days	[21]
MCL, DLBCL, FL	NCT02413489, CARINA	2	Dara single agent	Terminated as futility thresholds were not reached (FL ORR 50%), (DLBCL ORR 30%)	[22]
T ALL, TLBL	NCT02999633	2	Isa single agent	Terminated; unsatisfactory benefit/risk ratio, 11/14 developed progressive disease as best response.	[92]

Dara, daratumumab; Len, lenalidomide; Dex, dexamethasone; Pom, pomalidomide; Bort, bortezomib; Isa, isatuximab, Carfilz. Carfilzomib PFS, progression-free survival; ORR, overall response rate; TLBL, T-cell lymphoblastic lymphoma.

A total of 11 out of 14 patients developed progressive disease, which was ultimately the best response observed. Combination-based regimens containing CD38 antibodies are currently the subject of ongoing clinical trials in AML (NCT03537599), ALL (NCT03860844), and CLL (NCT04230304). In CLL, the phase 2 trial seeks to characterize the potential clinical efficacy of daratumumab in combination with ibrutinib, a BTK kinase which has become an established treatment for the relapsed/refractory disease. Analogous to the triplet drug combination success in MM, clinical evaluation of CD38 monoclonal antibodies in combination with drugs that rationally target key oncogenic pathways in that subtype of cancer may pave the way forward for improving patient response in hematological malignancies or daratumumab refractory patients.

## 4. Molecular Strategies to Enhance CD38 Expression for More Effective Targeting by Monoclonal Antibodies

Extensive studies in hematological malignancies highlight that, beyond simply a surface biomarker, CD38 transduces pathways which can promote tumor survival, expansion, and metastasis [18,66,68]. Thus, CD38 is an attractive and novel target, especially in highly aggressive hematologic malignancies for which novel treatment modalities are scarce. However, early-phase clinical trials with CD38 antibodies in some of these malignancies (Table 2) have only demonstrated limited efficacy so far. 

Considerable intra-tumoral heterogeneity in CD38 expression may be one of the key factors underlying the limited clinical efficacy of single-agent CD38 antibody treatment in some blood malignancies. In multiple myeloma, CD38 expression is typically high and homogenous, thereby supporting a sustainable therapeutic response [32]. CD38 expression, however, is more variable in CLL, where CD38 positivity is defined as ≥20% expression [94]. In the phase 2 daratumumab trial in NKTL, half of the patient cohort exhibited less than 50% CD38 expression [21]. Thus, administration of CD38 monoclonal antibodies may selectively deplete CD38-positive cells, possibly enriching the CD38-negative population, which subsequently emerges as treatment-resistant clones. In the phase 2 trial of isatuximab in T-ALL and T-LBL, CD38 expression was uniformly high across leukemic blasts at all stages of the disease. This is, however, not as high as levels observed in MM, which suggests that achieving a minimal threshold for CD38 expression may reverse poor outcomes in this study [92]. 

**Figure 3:**
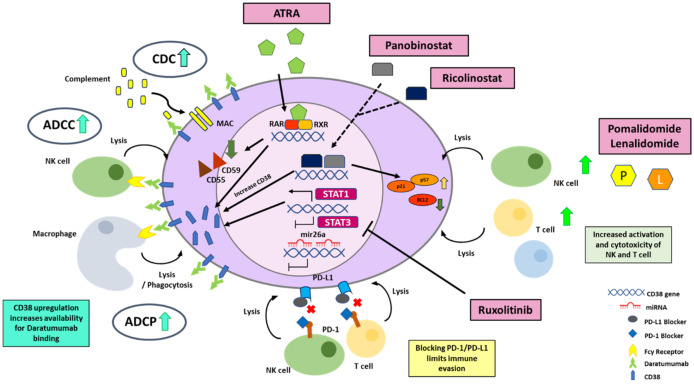
Molecular strategies to enhance CD38 expression and overall antitumor efficacy of Daratumumab. A. Transcriptional upregulation of the CD38 mRNA and subsequent protein expression can be stimulated by ATRA, HDAC inhibitors (panobinostat and ricolinostat), STAT3 inhibitor (ruxolitinib) and immunomodulatory drugs (pomalidomide and lenalidomide). B. CD38 mRNA can be degraded by miR-26a and miR-140-3p through direct binding to the 3’UTR or indirectly through the cytokine mediated mechanisms. These miRs can be targeted through antisense oligonucleotides to prevent CD38 mRNA degradation. C. Optimization of CD38 availability on the cell surface membrane by modulating processes involved in extracellular vesicle formation or trogocytosis.

Thus, to overcome these limiting factors, incorporation of strategies that amplify CD38 surface expression may allow us to fully harness the antitumor potential of CD38 antibodies. In the following section, we will describe molecular modulators of CD38 expression and discuss how these can be exploited to upregulate CD38 levels to enhance therapeutic efficacies (Figure 3).

### 4.1. The Human CD38 Gene

Briefly, the human CD38 is located on the short arm of chromosome 4 (4p15) and exhibits several distinctive features. First, the gene is relatively large, with >98% of the genetic material consisting of introns. The first intron of the CD38 gene contains a retinoic acid response element (RARE), which can be bound by a heterodimer composed of RA receptor and retinoid X receptor in vitro [95]. Indeed, induction of the transcriptional upregulation of CD38 through retinoids have been well described. The promoter region of CD38 is atypical, lacking a canonical TATA box but containing a CpG island, thereby suggesting susceptibility to epigenetic modulators, which has been increasingly observed in cancer studies [96]. The methylation of this CpG island on the CD38 promoter has been shown to negatively correlate with surface expression in CLL patients [97]. CD38 expression in PBMCs is under the pressure of constant and intense regulatory activity. Retinoids, vitamin D, and a variety of cytokines are the most known inducers [32]. 

### 4.2. Strategies to Enhance Transcriptional Activation of CD38 Gene 

#### 4.2.1. All Trans Retinoic Acid (ATRA)

The ATRA molecule binds to nuclear retinoic acid receptor (RAR), which then mediates upregulation of CD38 mRNA expression through RARE [98,99], subsequently leading to enhanced CD38 surface protein expression. Thus, ATRA can be utilized to boost CD38 levels, particularly in patients with low CD38 expression to enhance therapeutic efficacy of CD38 monoclonal antibodies. 

Preclinical evaluation of this strategy has confirmed that ATRA can significantly enhance CD38 expression and trigger overall amplification of Fc-receptor-dependent effector mechanisms ADCC and CDC in a variety of cancer models [14,100]. Treatment of MM cell lines with Daratumumab, after ATRA exposure, improved ADCC and CDC lysis, which correlated with the upregulation of CD38 in both MM cell lines and patient samples. It is interesting to note that the increase in CDC lysis is significantly higher than the increase in ADCC, possibly due to the downregulation of the complement inhibitory proteins via ATRA exposure [100]. Van de Donk and his colleagues further evaluated the efficacy and safety of daratumumab combined with ATRA in daratumumab refractory MM patients. Although 66% of patients achieved stable disease, the primary endpoint was not met, with an overall response rate of 5% [101]. Although ATRA increased CD38 expression, limited efficacy was observed, potentially because this effect was transient and not able to restore CD38 expression to baseline levels. 

This combination has yet to be evaluated clinically for other blood cancers. Preclinical studies have shown that ATRA-mediated CD38 upregulation can also enhance the immunomodulatory effects mediated by CD38 in AML and CML via reversal of tumor migration and NAD+ mediated resistance mechanisms, respectively [83,102].

#### 4.2.2. HDAC Inhibitors

Another possible way to upregulate CD38 expression is via the use of histone deacetylase inhibitor, Panobinostat, although the mechanism of upregulation is not quite clear. Panobinostat has been shown to increase CD38 expression in primary MM cells and MM cell lines [103]. Upon stopping treatment, CD38 levels reverted back to baseline level and then, upon re-exposure of Panobinostat, CD38 upregulation returned to levels similar to that of the primary treatment. Moreover, Daratumumab treatment after Panobinostat pretreatment led to higher ADCC in both MM cell lines and primary cells [103]. Interestingly, this upregulation in CD38 expression levels was only observed in myeloma cells, not in T cells, and also lymphoma cell lines. Apart from CD38 upregulation, Panobinostat exposure has led to several changes in transcriptional profile of myeloma cells, which may aid in overcoming drug resistance in MM, making it a promising candidate for drug combination treatments. It was observed to inhibit cell cycle progression and associated with upregulation of p21, p53, and p57. It also promotes apoptosome formation along with Apaf-1 upregulation and downregulation of antiapoptotic proteins, such as BCL2 and BCL XL [104]. Panobinostat also induces histone H4 acetylation, which may lead to activation of tumor suppressor genes. On top of that, it has been associated with activation of caspases, which eventually lead to apoptotic cell death. In vitro studies have also shown decreased viability of MM cell lines when used in combination with Melphalan or Doxorubicin [105]. 

Furthermore, there has been a recent report highlighting a basal role for HDAC6-mediated promoter deacetylation in regulating CD38 gene expression levels in MM [106]. Addition of Ricolinostat to patient MM cells resulted in the upregulation of CD38 mRNA transcripts and subsequent enhancement of CD38 surface expression. Chromatin immunoprecipitation assays performed after Ricolinostat treatment confirmed an increased acetylation of H3K27 at the CD38 promoter and, hence, the activation of the gene. Similar to Panobinostat, Ricolinostat only enhances CD38 expression in MM and not T or NK cells, thus evading any off-target augmentation in tumor suppression by CD38 T-regulatory populations. HDAC inhibitors have been shown to suppress myeloid-derived suppressor cells, another potent immune suppressive cell population [107], thereby underscoring the potentially impactful therapeutic effect induced by the combination of Ricolinostat and daratumumab.

#### 4.2.3. STAT 3 Inhibitors

The bone marrow niche in MM is an integral component of the pro-tumoral microenvironment sustaining and advancing the cancer. Bone marrow stromal factors modulating the expression of CD38 and ADCC effector mechanisms of daratumumab were studied in depth and this led to the discovery of the converse roles mediated by the IL6-induced JAK STAT pathway in MM [108]. JAK-STAT3 signaling was found to suppress CD38 expression, whereas the JAK-STAT1 pathway mediated CD38 upregulation. This was further corroborated with the inverse correlation between STAT 3 and CD38 expression in MM patient cells. Ruxolitinib a Jak1/2 selective inhibitor, which inhibits the phosphorylation of both STAT3 and STAT1 overall mediated a transcriptional upregulation of the CD38 mRNA transcript in some patients. Its effect is less consistent than ATRA and this may be attributable to the concomitant inhibition of STAT1, which is a promoter of CD38 expression. Nonetheless, Ruxolitinib can still rescue the BM-supernatant-mediated loss of CD38 surface expression and suppression of ADCC on patient MM cells [108]. Selective STAT3 inhibitors or STAT1 activators are potential candidates, which can be evaluated in the future in combination with daratumumab treatment.

#### 4.2.4. Immunomodulatory Imide Drugs (IMiDs)

First-in-class immunomodulatory drugs (IMiDs) lenalidomide and pomalidomide currently form the cornerstone of MM therapy. The promising efficacy of IMiDs and CD38-targeting therapeutics was first highlighted in 2016 by the phase 3 POLLUX clinical trial in relapsed refractory MM patients [85]. This study demonstrated a significant superior overall response rate (93% vs. 76%) and progression-free survival (median 44.5 vs. 17.5 months) in patients treated with a combination of daratumumab–lenalidomide–dexamethasone as compared to lenalidomide and dexamethasone only [85,86]. One of the mechanisms underlying this synergistic efficacy appears to be IMiD-mediated upregulation of CD38 expression. Both lenalidomide and pomalidomide have been shown to stimulate the increase in CD38 surface expression, with the latter demonstrating up to threefold upregulation at 72 h in vitro [109,110,111]. This appears to be triggered by the IMiD-mediated degradation of Ikaros and Aiolos. In MM, Ikaros interacts with CHD4, a component of the nucleosome remodeling deacetylase complex (NurD), to repress CD38 mRNA expression [112]. Depletion of Ikaros and Aiolos via Crispr-Cas9 knockout or IMiD treatment releases this suppression and stimulates transcriptional upregulation of CD38 mRNA, which subsequently primes the cells for enhanced daratumumab-induced ADCC [112]. This elegantly provides a cell intrinsic mechanism explaining the clinically favorable outcomes for the treatment combination of IMiDs and daratumumab.

### 4.3. Strategies Regulating the Degradation of CD38 mRNA

Another interesting component of CD38 regulation that has emerged recently is the post-transcriptional regulation of CD38 expression by microRNAs. MicroRNAs are a family of short noncoding RNAs that generally promote mRNA decay and degradation via binding to the 3′ untranslated regions, thereby resulting in gene silencing. In human cancers, miRNAs may function as either oncogenes or tumor suppressors to regulate pathways that promote proliferation, invasion, and metastasis [113]. MiRNAs are also increasingly established as potential biomarkers for human cancer diagnosis and prognosis, as well as therapeutic targets or tools in the clinic. Little is known about the posttranscriptional regulation of CD38 mRNA. Here, we describe two studies identifying miRNA candidates that can modulate CD38 expression.

MiR-26a expression was found to be significantly downregulated in the plasma cells of multiple myeloma patients, as compared to healthy donors. The direct binding of miR-26a to the 3′ UTR of the CD38 mRNA inhibited the expression of CD38 protein in MM cell lines [114]. MiR-26a-mediated downregulation of CD38 expression was able to trigger apoptosis. This was further validated in an MM xenograft model where miR-26a mimics not only inhibited CD38 expression, but also reduced proliferation and induced caspase-3 cleavage in vivo. It will be interesting to evaluate the levels of mir-26a in patients along the course of the daratumumab treatment and to study if CD38 expression can be optimized through the utilization of miR-26a mimics or antisense oligonucleotides. The modulation of CD38 expression levels to achieve potentially optimal levels may resensitize resistant cells to daratumumab treatment, thereby deriving some clinical benefit.

miRNA-mediated regulation of CD38 has also been reported in human airway smooth muscle cells. Here, TNF-alpha-induced miR-140-3p modulation of CD38 expression was mediated directly through binding to the CD38 3′ UTR and indirectly through cytokine mechanisms involving activation of p38 MAPK and the transcription factor NF-kB [115].

### 4.4. Strategies to Optimize CD38 Antigen Availability on the Cell Membrane

Finally, at the membrane protein surface expression level, the availability of the CD38 antigen to mediate anticancer mechanisms stimulated by the CD38 monoclonal antibodies has been shown to be a limiting factor over time. Daratumumab infusion induces a quick and consistent reduction in CD38 on MM cells, with up to an estimated 90% reduction at the first infusion [116,117]. There are several proposed mechanisms leading to this. Firstly, daratumumab treatment selectively lyses high CD38-expressing MM cells via ADCC and CDC, thereby enriching the population of low CD38-expressing MM cells and facilitating its clonal expansion [118]. Secondly, binding of daratumumab can lead to the internalization of the protein through endocytosis [119,120]. Thirdly, the CD38-daratumumab complex is shown to be transferred from MM cells to monocytes and granulocytes in a process known as trogocytosis, even with the absence of phagocytosis of tumor cells [116]. Trogocytosis is characterized by the process whereby chelated ligands on the donor cell, along with sections of its plasma membrane being pinched off and taken up by the acceptor cell [121]. Lastly, CD38 surface protein depletion may be a result of the redistribution of CD38 molecules upon daratumumab binding leading to the formation of polar aggregates, which are subsequently released as microvesicles (MV) [122,123]. CD38 expression on these microvesicles is colocalized with CD73, CD39, and CD203, which functionally points to potential microvesicle-mediated production of adenosine through this complex and the maintenance of a tolerogenic environment [123]. The microvesicles also carry PDL 1 and CD55 and CD59. In this context, the loss of CD38 expression to the MVs seems to be a way for the cancer to overcome CD38 therapeutic effects. 

It is interesting to note that there was also a study published recently describing the involvement of extracellular vesicles (EV) in daratumumab-resistant models, which have been exposed to sequentially increasing concentrations of daratumumab. In RNA sequencing analyses made, there was an enrichment for genes involved in exosome biogenesis and secretion in both cell line and mouse-derived daratumumab-resistant models of NKTL [124]. This was further validated in additional Dara refractory models of myeloma and T-ALL, which displayed increased concentration of secreted exosome particles and expression of exosome markers in the media [124]. Inhibition of these isogenic daratumumab-sensitive and -resistant cell line pairs with inhibitors of exosome secretion demonstrated a selective and more effective suppression of tumor cell viability in daratumumab-resistant than -sensitive cell lines, suggesting an addiction to the exosome biogenesis pathway for survival. These studies suggest that the role of extracellular vesicles in mediating resistance to daratumumab may be mitigated by inhibitors of EV formation and that this line of investigation may yield alternative novel combinations to enhance the efficacy of daratumumab treatment. 

## 5. Conclusions and Future Directions

A lot of studies are focused on the optimization of CD38-targeting antibodies in multiple myeloma, as well as the elucidation of the detailed mechanism of action from the point of patient exposure to the development of a refractory response, and rightly so. Mechanistic insights to antitumoral mechanisms mediated by CD38 antibodies in multiple myeloma are a model to be learnt from and, most importantly, applied and extended to other CD38-dependent malignancies or diseases. The tumorigenic roles of CD38 in myeloma and other hematologic malignancies were highlighted in this review so as to gain a better insight as to the various determinants of response that can be further targeted to enhance CD38-directed antitumor mechanisms. 

Apart from amplifying CD38 surface expression, there are other factors that can enhance CD38-targeting therapies. For example, the synergistic combination of daratumumab and IMiDs can also trigger immune stimulatory activities, which potentiates the cytotoxic effects of T and NK cells [125] on tumor cells, thereby promoting enhanced ADCC and T-cell lysis [126,127]. In the presence of IMiDs, isatuximab also induces enhanced direct cell apoptosis, augmented PBMC-mediated ADCC in MM cell lines and patient samples, and CD38 expression on Tregs was decreased, thus affording a further relief from the immunosuppressive tumor niche [7]. Interestingly, patients who were initially refractory to an IMiD drug in a prior line of therapy was resensitized to lenalidomide or pomalidomide after starting on daratumumab [128]. Blocking the PD-1/PDL-1 axis to prevent tumor evasion is also another combination that is currently being explored with daratumumab. A preclinical study conducted to measure the efficacy of Daratumumab and anti-PD-1 (Nivolumab) combination treatment on a mouse tumor model demonstrated prolonged survival and enhanced tumor regression [129]. 

The clinical success of CD38 monoclonal antibodies in MM has stimulated the development of cytotoxic T-cell-based therapeutics, such as CD38 chimeric antigen receptor (CAR) T cells and CD38 (bispecific T-cell engager) BiTE antibodies. BCMA and CD19 CAR T treatment has been approved in DLBCL, ALL, and MM, thereby highlighting the potential clinical utility of a CD38 CAR T treatment [130,131]. The CD38 CAR T stably expresses a single chain fragment variant that recognizes the CD38 antigen and subsequently triggers cytotoxic T-cell responses through the activating of cytoplasmic domain upon binding. Preclinical evaluation of CD38 CAR T in MM, AML, NKTL, and MCL suggests that this can effectively suppress CD38hi-expressing subsets of the cancer [130]. Combination of CD38 CAR T with CD38 transcriptional activators, such as ATRA, further improve therapeutic efficacy in mouse xenografts, emphasizing the importance of optimal induction of CD38 expression in cancer cells. Designing tandem dual CAR Ts, which targets multiple antigens simultaneously, can increase specificity of effector cells and potentially circumvent to antigen escape [132]. Clinical trials are ongoing to evaluate the single dose escalation safety and efficacy outcomes of CD38 CAR-T-cell therapy in relapsed or refractory MM patients (NCT03464916 and NCT03767751). In a phase 1/2 trial for CD38 CAR T in AML, the median leukemia-free survival (LFS) time was 6.4 months with manageable side effects [133]. 

CD38 bispecific T-cell-engaging (BiTE) antibodies belong to a new class of immunotherapeutic agents, which can mediate dual specific binding to the CD38 and the CD3e chain on T cells, thereby activating T cells and recruiting them in proximity of target cancer cells to efficiently induce T-cell-mediated cytotoxicity. One such antibody, Bi38-3, can suppress MM growth both in vitro and in vivo without reducing surface expression of CD38 [134]. T-cell-mediated cytotoxic responses are only induced in CD38hi tumor cells with limited toxicity against cells expressing intermediate levels of CD38. This suggests yet again that CD38 surface expression may present as the biggest limiting factor for CD38 therapeutic efficacy in blood cancers with heterogenous expression of CD38. 

Strategies enhancing the transcriptional, translational, or protein surface regulation of CD38 should be seriously evaluated. In light of recent findings, unique and novel treatment combinations incorporating modulators of CD38 expression, including HDAC6 inhibitors, STAT 3/1 modulators, miRNA mimics, or antisense oligonucleotides, as well as agents regulating formation and secretion of extracellular vesicles, should be extensively considered to be investigated in combination with CD38 antibodies.

Additionally, several recent reviews have highlighted that, despite excellent treatment outcomes, there remains a subset of patients who eventually develop resistance to daratumumab [135,136]. Here, CD38 expression levels may have less impact and strategies that may potentially overcome refractoriness to daratumumab include treatments which can reverse NK cell depletion, for example, through the transfusion of ex vivo expanded NK cells [118,137,138], countering the increased expression of complement inhibitory proteins during disease progression with CD55- or CD59-blocking antibodies [117,139] and potentially utilizing anti-CD38 antibody-drug conjugates to enhance the cytotoxic load to the cells [118,140].

## Data Availability

Not applicable.

## References

[1] Liu J.K. (2014). The history of monoclonal antibody development—Progress, remaining challenges and future innovations. Ann. Med. Surg..

[2] Melero I., Hervas-Stubbs S., Glennie M., Pardoll D.M., Chen L. (2007). Immunostimulatory monoclonal antibodies for cancer therapy. Nat. Rev. Cancer.

[3] Mateos M.V., Dimopoulos M.A., Cavo M., Suzuki K., Jakubowiak A., Knop S., Doyen C., Lucio P., Nagy Z., Kaplan P. (2018). Daratumumab plus Bortezomib, Melphalan, and Prednisone for Untreated Myeloma. N. Engl. J. Med..

[4] Moreau P., Attal M., Hulin C., Arnulf B., Belhadj K., Benboubker L., Bene M.C., Broijl A., Caillon H., Caillot D. (2019). Bortezomib, thalidomide, and dexamethasone with or without daratumumab before and after autologous stem-cell transplantation for newly diagnosed multiple myeloma (CASSIOPEIA): A randomised, open-label, phase 3 study. Lancet.

[5] Palumbo A., Chanan-Khan A., Weisel K., Nooka A.K., Masszi T., Beksac M., Spicka I., Hungria V., Munder M., Mateos M.V. (2016). Daratumumab, Bortezomib, and Dexamethasone for Multiple Myeloma. N. Engl. J. Med..

[6] Postigo J., Iglesias M., Cerezo-Wallis D., Rosal-Vela A., García-Rodríguez S., Zubiaur M., Sancho J., Merino R., Merino J. (2012). Mice Deficient in CD38 Develop an Attenuated Form of Collagen Type II-Induced Arthritis. PLoS ONE.

[7] Jiang H., Acharya C., An G., Zhong M., Feng X., Wang L., Dasilva N., Song Z., Yang G., Adrian F. (2016). SAR650984 directly induces multiple myeloma cell death via lysosomal-associated and apoptotic pathways, which is further enhanced by pomalidomide. Leukemia.

[8] van Bueren J.L., Jakobs D., Kaldenhoven N., Roza M., Hiddingh S., Meesters J., Voorhorst M., Gresnigt E., Wiegman L., Buijsse A.O. (2014). Direct in Vitro Comparison of Daratumumab with Surrogate Analogs of CD38 Antibodies MOR03087, SAR650984 and Ab79. Blood.

[9] Richardson P.G., Perrot A., San-Miguel J., Beksac M., Spicka I., Leleu X., Schjesvold F., Moreau P., Dimopoulos M.A., Huang J.S. (2022). Isatuximab plus pomalidomide and low-dose dexamethasone versus pomalidomide and low-dose dexamethasone in patients with relapsed and refractory multiple myeloma (ICARIA-MM): Follow-up analysis of a randomised, phase 3 study. Lancet Oncol..

[10] Moreau P., Dimopoulos M.A., Mikhael J., Yong K., Capra M., Facon T., Hajek R., Spicka I., Baker R., Kim K. (2021). Isatuximab, carfilzomib, and dexamethasone in relapsed multiple myeloma (IKEMA): A multicentre, open-label, randomised phase 3 trial. Lancet.

[11] Raab M.S., Engelhardt M., Blank A., Goldschmidt H., Agis H., Blau I.W., Einsele H., Ferstl B., Schub N., Rollig C. (2020). MOR202, a novel anti-CD38 monoclonal antibody, in patients with relapsed or refractory multiple myeloma: A first-in-human, multicentre, phase 1-2a trial. Lancet Haematol..

[12] Fedyk E.R., Zhao L., Koch A., Smithson G., Estevam J., Chen G., Lahu G., Roepcke S., Lin J., McLean L. (2020). Safety, tolerability, pharmacokinetics and pharmacodynamics of the anti-CD38 cytolytic antibody TAK-079 in healthy subjects. Br. J. Clin. Pharmacol..

[13] Wada F., Shimomura Y., Yabushita T., Yamashita D., Ohno A., Imoto H., Maruoka H., Hara S., Ishikawa T. (2021). CD38 expression is an important prognostic marker in diffuse large B-cell lymphoma. Hematol. Oncol..

[14] Mustafa N., Nee A.H.F., Chooi J.Y., Toh S.H.M., Chung T.H., Selvarajan V., Fan S., Ng S.B., Poon M., Chan E. (2021). Determinants of response to daratumumab in Epstein-Barr virus-positive natural killer and T-cell lymphoma. J. Immunother. Cancer.

[15] Bride K.L., Vincent T.L., Im S.Y., Aplenc R., Barrett D.M., Carroll W.L., Carson R., Dai Y., Devidas M., Dunsmore K.P. (2018). Preclinical efficacy of daratumumab in T-cell acute lymphoblastic leukemia. Blood.

[16] Ghia P., Guida G., Stella S., Gottardi D., Geuna M., Strola G., Scielzo C., Caligaris-Cappio F. (2003). The pattern of CD38 expression defines a distinct subset of chronic lymphocytic leukemia (CLL) patients at risk of disease progression. Blood.

[17] Damle R.N., Wasil T., Fais F., Ghiotto F., Valetto A., Allen S.L., Buchbinder A., Budman D., Dittmar K., Kolitz J. (1999). Ig V gene mutation status and CD38 expression as novel prognostic indicators in chronic lymphocytic leukemia. Blood.

[18] Deaglio S., Vaisitti T., Bergui L., Bonello L., Horenstein A.L., Tamagnone L., Boumsell L., Malavasi F. (2005). CD38 and CD100 lead a network of surface receptors relaying positive signals for B-CLL growth and survival. Blood.

[19] Vaisitti T., Aydin S., Rossi D., Cottino F., Bergui L., D’Arena G., Bonello L., Horenstein A.L., Brennan P., Pepper C. (2010). CD38 increases CXCL12-mediated signals and homing of chronic lymphocytic leukemia cells. Leukemia.

[20] Horenstein A.L., Quarona V., Toscani D., Costa F., Chillemi A., Pistoia V., Giuliani N., Malavasi F. (2016). Adenosine Generated in the Bone Marrow Niche Through a CD38-Mediated Pathway Correlates with Progression of Human Myeloma. Mol. Med..

[21] Huang H., Zhu J., Yao M., Kim T.M., Yoon D.H., Cho S.G., Eom H.S., Lim S.T., Yeh S.P., Song Y. (2021). Daratumumab monotherapy for patients with relapsed or refractory natural killer/T-cell lymphoma, nasal type: An open-label, single-arm, multicenter, phase 2 study. J. Hematol. Oncol..

[22] Salles G., Gopal A.K., Minnema M.C., Wakamiya K., Feng H., Schecter J.M., Wang M. (2019). Phase 2 Study of Daratumumab in Relapsed/Refractory Mantle-Cell Lymphoma, Diffuse Large B-Cell Lymphoma, and Follicular Lymphoma. Clin. Lymphoma Myeloma Leuk..

[23] Reinherz E.L., Kung P.C., Goldstein G., Levey R.H., Schlossman S.F. (1980). Discrete stages of human intrathymic differentiation: Analysis of normal thymocytes and leukemic lymphoblasts of T-cell lineage. Proc. Natl. Acad. Sci. USA.

[24] Lee H.C. (2006). Structure and enzymatic functions of human CD38. Mol. Med..

[25] Lund F., Solvason N., Grimaldi J.C., Parkhouse R.M., Howard M. (1995). Murine CD38: An immunoregulatory ectoenzyme. Immunol. Today.

[26] Malavasi F., Funaro A., Roggero S., Horenstein A., Calosso L., Mehta K. (1994). Human CD38: A glycoprotein in search of a function. Immunol. Today.

[27] Mizuguchi M., Otsuka N., Sato M., Ishii Y., Kon S., Yamada M., Nishina H., Katada T., Ikeda K. (1995). Neuronal localization of CD38 antigen in the human brain. Brain Res..

[28] Cockayne D.A., Muchamuel T., Grimaldi J.C., Muller-Steffner H., Randall T.D., Lund F.E., Murray R., Schuber F., Howard M.C. (1998). Mice deficient for the ecto-nicotinamide adenine dinucleotide glycohydrolase CD38 exhibit altered humoral immune responses. Blood.

[29] Nishina H., Inageda K., Takahashi K., Hoshino S., Ikeda K., Katada T. (1994). Cell surface antigen CD38 identified as ecto-enzyme of NAD glycohydrolase has hyaluronate-binding activity. Biochem. Biophys. Res. Commun..

[30] Newman P.J. (1999). Switched at birth: A new family for PECAM-1. J. Clin. Investig..

[31] Frasca L., Fedele G., Deaglio S., Capuano C., Palazzo R., Vaisitti T., Malavasi F., Ausiello C.M. (2006). CD38 orchestrates migration, survival, and Th1 immune response of human mature dendritic cells. Blood.

[32] Malavasi F., Deaglio S., Funaro A., Ferrero E., Horenstein A.L., Ortolan E., Vaisitti T., Aydin S. (2008). Evolution and function of the ADP ribosyl cyclase/CD38 gene family in physiology and pathology. Physiol. Rev..

[33] Deterre P., Berthelier V., Bauvois B., Dalloul A., Schuber F., Lund F. (2000). CD38 in T- and B-cell functions. Chem. Immunol..

[34] Howard M., Grimaldi J.C., Bazan J.F., Lund F.E., Santos-Argumedo L., Parkhouse R.M., Walseth T.F., Lee H.C. (1993). Formation and hydrolysis of cyclic ADP-ribose catalyzed by lymphocyte antigen CD38. Science.

[35] Zocchi E., Franco L., Guida L., Benatti U., Bargellesi A., Malavasi F., Lee H.C., De Flora A. (1993). A single protein immunologically identified as CD38 displays NAD+ glycohydrolase, ADP-ribosyl cyclase and cyclic ADP-ribose hydrolase activities at the outer surface of human erythrocytes. Biochem. Biophys. Res. Commun..

[36] Lee H.C., Aarhus R. (1995). A derivative of NADP mobilizes calcium stores insensitive to inositol trisphosphate and cyclic ADP-ribose. J. Biol. Chem..

[37] Schuber F., Lund F.E. (2004). Structure and enzymology of ADP-ribosyl cyclases: Conserved enzymes that produce multiple calcium mobilizing metabolites. Curr. Mol. Med..

[38] Horenstein A.L., Chillemi A., Zaccarello G., Bruzzone S., Quarona V., Zito A., Serra S., Malavasi F. (2013). A CD38/CD203a/CD73 ectoenzymatic pathway independent of CD39 drives a novel adenosinergic loop in human T lymphocytes. Oncoimmunology.

[39] Ramakers B.P., Wever K.E., Kox M., van den Broek P.H., Mbuyi F., Rongen G., Masereeuw R., van der Hoeven J.G., Smits P., Riksen N.P. (2012). How systemic inflammation modulates adenosine metabolism and adenosine receptor expression in humans in vivo. Crit. Care Med..

[40] Horenstein A.L., Bracci C., Morandi F., Malavasi F. (2019). CD38 in Adenosinergic Pathways and Metabolic Re-programming in Human Multiple Myeloma Cells: In-tandem Insights From Basic Science to Therapy. Front. Immunol..

[41] de Weers M., Tai Y.T., van der Veer M.S., Bakker J.M., Vink T., Jacobs D.C., Oomen L.A., Peipp M., Valerius T., Slootstra J.W. (2011). Daratumumab, a novel therapeutic human CD38 monoclonal antibody, induces killing of multiple myeloma and other hematological tumors. J. Immunol..

[42] Overdijk M.B., Jansen J.H., Nederend M., Lammerts van Bueren J.J., Groen R.W., Parren P.W., Leusen J.H., Boross P. (2016). The Therapeutic CD38 Monoclonal Antibody Daratumumab Induces Programmed Cell Death via Fcgamma Receptor-Mediated Cross-Linking. J. Immunol..

[43] van de Donk N.W., Janmaat M.L., Mutis T., Lammerts van Bueren J.J., Ahmadi T., Sasser A.K., Lokhorst H.M., Parren P.W. (2016). Monoclonal antibodies targeting CD38 in hematological malignancies and beyond. Immunol. Rev..

[44] Krejcik J., Casneuf T., Nijhof I.S., Verbist B., Bald J., Plesner T., Syed K., Liu K., van de Donk N.W., Weiss B.M. (2016). Daratumumab depletes CD38+ immune regulatory cells, promotes T-cell expansion, and skews T-cell repertoire in multiple myeloma. Blood.

[45] Gertz M.A., Kyle R.A., Greipp P.R. (1989). The plasma cell labeling index: A valuable tool in primary systemic amyloidosis. Blood.

[46] Muchtar E., Jevremovic D., Dispenzieri A., Dingli D., Buadi F.K., Lacy M.Q., Gonsalves W., Hayman S.R., Kapoor P., Leung N. (2017). The prognostic value of multiparametric flow cytometry in AL amyloidosis at diagnosis and at the end of first-line treatment. Blood.

[47] Kaufman G.P., Schrier S.L., Lafayette R.A., Arai S., Witteles R.M., Liedtke M. (2017). Daratumumab yields rapid and deep hematologic responses in patients with heavily pretreated AL amyloidosis. Blood.

[48] Di Nora C., Sponga S., Ferrara V., Patriarca F., Fanin R., Nalli C., Lechiancole A., Vendramin I., Livi U. (2021). Emerging therapy in light-chain and acquired transthyretin-related amyloidosis: An Italian single-centre experience in heart transplantation. J. Cardiovasc. Med..

[49] Morice W.G., Chen D., Kurtin P.J., Hanson C.A., McPhail E.D. (2009). Novel immunophenotypic features of marrow lymphoplasmacytic lymphoma and correlation with Waldenstrom’s macroglobulinemia. Mod. Pathol..

[50] San Miguel J.F., Vidriales M.B., Ocio E., Mateo G., Sanchez-Guijo F., Sanchez M.L., Escribano L., Barez A., Moro M.J., Hernandez J. (2003). Immunophenotypic analysis of Waldenstrom’s macroglobulinemia. Semin. Oncol..

[51] Konoplev S., Medeiros L.J., Bueso-Ramos C.E., Jorgensen J.L., Lin P. (2005). Immunophenotypic profile of lymphoplasmacytic lymphoma/Waldenstrom macroglobulinemia. Am. J. Clin. Pathol..

[52] (2020). Waldenström Macroglobulinemia Patients with Daratumumab. Clinicaltrials.gov.

[53] Hayakawa K., Esposito E., Wang X., Terasaki Y., Liu Y., Xing C., Ji X., Lo E.H. (2016). Transfer of mitochondria from astrocytes to neurons after stroke. Nature.

[54] Marlein C.R., Piddock R.E., Mistry J.J., Zaitseva L., Hellmich C., Horton R.H., Zhou Z., Auger M.J., Bowles K.M., Rushworth S.A. (2019). CD38-Driven Mitochondrial Trafficking Promotes Bioenergetic Plasticity in Multiple Myeloma. Cancer Res..

[55] Morandi F., Horenstein A.L., Chillemi A., Quarona V., Chiesa S., Imperatori A., Zanellato S., Mortara L., Gattorno M., Pistoia V. (2015). CD56brightCD16- NK Cells Produce Adenosine through a CD38-Mediated Pathway and Act as Regulatory Cells Inhibiting Autologous CD4+ T Cell Proliferation. J. Immunol..

[56] Karakasheva T.A., Waldron T.J., Eruslanov E., Kim S.B., Lee J.S., O’Brien S., Hicks P.D., Basu D., Singhal S., Malavasi F. (2015). CD38-Expressing Myeloid-Derived Suppressor Cells Promote Tumor Growth in a Murine Model of Esophageal Cancer. Cancer Res..

[57] Li M.O., Rudensky A.Y. (2016). T cell receptor signalling in the control of regulatory T cell differentiation and function. Nat. Rev. Immunol..

[58] Feng X., Zhang L., Acharya C., An G., Wen K., Qiu L., Munshi N.C., Tai Y.T., Anderson K.C. (2017). Targeting CD38 Suppresses Induction and Function of T Regulatory Cells to Mitigate Immunosuppression in Multiple Myeloma. Clin. Cancer Res..

[59] Patten P.E., Buggins A.G., Richards J., Wotherspoon A., Salisbury J., Mufti G.J., Hamblin T.J., Devereux S. (2008). CD38 expression in chronic lymphocytic leukemia is regulated by the tumor microenvironment. Blood.

[60] Hamblin T.J., Orchard J.A., Ibbotson R.E., Davis Z., Thomas P.W., Stevenson F.K., Oscier D.G. (2002). CD38 expression and immunoglobulin variable region mutations are independent prognostic variables in chronic lymphocytic leukemia, but CD38 expression may vary during the course of the disease. Blood.

[61] Chang C.C., Cleveland R.P. (2002). Conversion of CD38 and/or myeloid-associated marker expression status during the course of B-CLL: Association with a change to an aggressive clinical course. Blood.

[62] Del Poeta G., Maurillo L., Venditti A., Buccisano F., Epiceno A.M., Capelli G., Tamburini A., Suppo G., Battaglia A., Del Principe M.I. (2001). Clinical significance of CD38 expression in chronic lymphocytic leukemia. Blood.

[63] Ibrahim S., Keating M., Do K.A., O’Brien S., Huh Y.O., Jilani I., Lerner S., Kantarjian H.M., Albitar M. (2001). CD38 expression as an important prognostic factor in B-cell chronic lymphocytic leukemia. Blood.

[64] Zucchetto A., Vaisitti T., Benedetti D., Tissino E., Bertagnolo V., Rossi D., Bomben R., Dal Bo M., Del Principe M.I., Gorgone A. (2012). The CD49d/CD29 complex is physically and functionally associated with CD38 in B-cell chronic lymphocytic leukemia cells. Leukemia.

[65] Zucchetto A., Benedetti D., Tripodo C., Bomben R., Dal Bo M., Marconi D., Bossi F., Lorenzon D., Degan M., Rossi F.M. (2009). CD38/CD31, the CCL3 and CCL4 chemokines, and CD49d/vascular cell adhesion molecule-1 are interchained by sequential events sustaining chronic lymphocytic leukemia cell survival. Cancer Res..

[66] Mele S., Devereux S., Pepper A.G., Infante E., Ridley A.J. (2018). Calcium-RasGRP2-Rap1 signaling mediates CD38-induced migration of chronic lymphocytic leukemia cells. Blood Adv..

[67] Deaglio S., Vaisitti T., Billington R., Bergui L., Omede P., Genazzani A.A., Malavasi F. (2007). CD38/CD19: A lipid raft-dependent signaling complex in human B cells. Blood.

[68] Krober A., Seiler T., Benner A., Bullinger L., Bruckle E., Lichter P., Dohner H., Stilgenbauer S. (2002). V(H) mutation status, CD38 expression level, genomic aberrations, and survival in chronic lymphocytic leukemia. Blood.

[69] Ottaggio L., Viaggi S., Zunino A., Zupo S., Rossi E., Spriano M., Abbondandolo A., Ferrarini M. (2003). Chromosome aberrations evaluated by comparative genomic hybridization in B-cell chronic lymphocytic leukemia: Correlation with CD38 expression. Haematologica.

[70] Smith A., Crouch S., Lax S., Li J., Painter D., Howell D., Patmore R., Jack A., Roman E. (2015). Lymphoma incidence, survival and prevalence 2004-2014: Sub-type analyses from the UK’s Haematological Malignancy Research Network. Br. J. Cancer.

[71] Dreyling M., Campo E., Hermine O., Jerkeman M., Le Gouill S., Rule S., Shpilberg O., Walewski J., Ladetto M., Committee E.G. (2017). Newly diagnosed and relapsed mantle cell lymphoma: ESMO Clinical Practice Guidelines for diagnosis, treatment and follow-up. Ann. Oncol..

[72] Espinet B., Ferrer A., Bellosillo B., Nonell L., Salar A., Fernandez-Rodriguez C., Puigdecanet E., Gimeno J., Garcia-Garcia M., Vela M.C. (2014). Distinction between asymptomatic monoclonal B-cell lymphocytosis with cyclin D1 overexpression and mantle cell lymphoma: From molecular profiling to flow cytometry. Clin. Cancer Res..

[73] Perez-Galan P., Mora-Jensen H., Weniger M.A., Shaffer A.L., Rizzatti E.G., Chapman C.M., Mo C.C., Stennett L.S., Rader C., Liu P. (2011). Bortezomib resistance in mantle cell lymphoma is associated with plasmacytic differentiation. Blood.

[74] Pfreundschuh M., Trumper L., Osterborg A., Pettengell R., Trneny M., Imrie K., Ma D., Gill D., Walewski J., Zinzani P.L. (2006). CHOP-like chemotherapy plus rituximab versus CHOP-like chemotherapy alone in young patients with good-prognosis diffuse large-B-cell lymphoma: A randomised controlled trial by the MabThera International Trial (MInT) Group. Lancet Oncol..

[75] Coiffier B., Thieblemont C., Van Den Neste E., Lepeu G., Plantier I., Castaigne S., Lefort S., Marit G., Macro M., Sebban C. (2010). Long-term outcome of patients in the LNH-98.5 trial, the first randomized study comparing rituximab-CHOP to standard CHOP chemotherapy in DLBCL patients: A study by the Groupe d’Etudes des Lymphomes de l’Adulte. Blood.

[76] Alsuwaidan A., Pirruccello E., Jaso J., Koduru P., Garcia R., Krueger J., Doucet M., Chaudhry R., Fuda F., Chen W. (2019). Bright CD38 Expression by Flow Cytometric Analysis Is a Biomarker for Double/Triple Hit Lymphomas with a Moderate Sensitivity and High Specificity. Cytom. B Clin. Cytom..

[77] Di Gaetano R., Gasparetto V., Padoan A., Callegari B., Candiotto L., Sanzari M.C., Scapinello A., Tagariello G. (2014). Flow cytometry CD4(+)CD26(-)CD38(+) lymphocyte subset in the microenvironment of Hodgkin lymphoma-affected lymph nodes. Ann. Hematol..

[78] Fox C.P., Civallero M., Ko Y.H., Manni M., Skrypets T., Pileri S., Kim S.J., Cabrera M.E., Shustov A.R., Chiattone C.S. (2020). Survival outcomes of patients with extranodal natural-killer T-cell lymphoma: A prospective cohort study from the international T-cell Project. Lancet Haematol..

[79] Zaja F., Tabanelli V., Agostinelli C., Calleri A., Chiappella A., Varettoni M., Luigi Zinzani P., Volpetti S., Sabattini E., Fanin R. (2017). CD38, BCL-2, PD-1, and PD-1L expression in nodal peripheral T-cell lymphoma: Possible biomarkers for novel targeted therapies?. Am. J. Hematol..

[80] Wang L., Wang H., Li P.F., Lu Y., Xia Z.J., Huang H.Q., Zhang Y.J. (2015). CD38 expression predicts poor prognosis and might be a potential therapy target in extranodal NK/T cell lymphoma, nasal type. Ann. Hematol..

[81] Naik J., Themeli M., de Jong-Korlaar R., Ruiter R.W.J., Poddighe P.J., Yuan H., de Bruijn J.D., Ossenkoppele G.J., Zweegman S., Smit L. (2019). CD38 as a therapeutic target for adult acute myeloid leukemia and T-cell acute lymphoblastic leukemia. Haematologica.

[82] Tembhare P.R., Sriram H., Khanka T., Chatterjee G., Panda D., Ghogale S., Badrinath Y., Deshpande N., Patkar N.V., Narula G. (2020). Flow cytometric evaluation of CD38 expression levels in the newly diagnosed T-cell acute lymphoblastic leukemia and the effect of chemotherapy on its expression in measurable residual disease, refractory disease and relapsed disease: An implication for anti-CD38 immunotherapy. J. Immunother. Cancer.

[83] Farber M., Chen Y., Arnold L., Mollmann M., Boog-Whiteside E., Lin Y.A., Reinhardt H.C., Duhrsen U., Hanoun M. (2021). Targeting CD38 in acute myeloid leukemia interferes with leukemia trafficking and induces phagocytosis. Sci. Rep..

[84] Muller K., Vogiatzi F., Winterberg D., Rosner T., Lenk L., Bastian L., Gehlert C.L., Autenrieb M.P., Bruggemann M., Cario G. (2022). Combining daratumumab with CD47 blockade prolongs survival in preclinical models of pediatric T-ALL. Blood.

[85] Dimopoulos M.A., Oriol A., Nahi H., San-Miguel J., Bahlis N.J., Usmani S.Z., Rabin N., Orlowski R.Z., Komarnicki M., Suzuki K. (2016). Daratumumab, Lenalidomide, and Dexamethasone for Multiple Myeloma. N. Engl. J. Med..

[86] Bahlis N.J., Dimopoulos M.A., White D.J., Benboubker L., Cook G., Leiba M., Ho P.J., Kim K., Takezako N., Moreau P. (2020). Daratumumab plus lenalidomide and dexamethasone in relapsed/refractory multiple myeloma: Extended follow-up of POLLUX, a randomized, open-label, phase 3 study. Leukemia.

[87] Dimopoulos M.A., Terpos E., Boccadoro M., Delimpasi S., Beksac M., Katodritou E., Moreau P., Baldini L., Symeonidis A., Bila J. (2021). Daratumumab plus pomalidomide and dexamethasone versus pomalidomide and dexamethasone alone in previously treated multiple myeloma (APOLLO): An open-label, randomised, phase 3 trial. Lancet Oncol..

[88] Martin T., Baz R., Benson D.M., Lendvai N., Wolf J., Munster P., Lesokhin A.M., Wack C., Charpentier E., Campana F. (2017). A phase 1b study of isatuximab plus lenalidomide and dexamethasone for relapsed/refractory multiple myeloma. Blood.

[89] van de Donk N., Richardson P.G., Malavasi F. (2018). CD38 antibodies in multiple myeloma: Back to the future. Blood.

[90] Avet-Loiseau H., San-Miguel J., Casneuf T., Iida S., Lonial S., Usmani S.Z., Spencer A., Moreau P., Plesner T., Weisel K. (2021). Evaluation of Sustained Minimal Residual Disease Negativity With Daratumumab-Combination Regimens in Relapsed and/or Refractory Multiple Myeloma: Analysis of POLLUX and CASTOR. J. Clin. Oncol..

[91] Usmani S.Z., Quach H., Mateos M.V., Landgren O., Leleu X., Siegel D., Weisel K., Gavriatopoulou M., Oriol A., Rabin N. (2022). Carfilzomib, dexamethasone, and daratumumab versus carfilzomib and dexamethasone for patients with relapsed or refractory multiple myeloma (CANDOR): Updated outcomes from a randomised, multicentre, open-label, phase 3 study. Lancet Oncol..

[92] Boissel N., Chevallier P., Doronin V., Griskevicius L., Maschan A., McCloskey J., Rambaldi A., Rossi G., Sokolov A., Wartiovaara-Kautto U. (2022). Isatuximab monotherapy in patients with refractory T-acute lymphoblastic leukemia or T-lymphoblastic lymphoma: Phase 2 study. Cancer Med..

[93] Spencer A., Lentzsch S., Weisel K., Avet-Loiseau H., Mark T.M., Spicka I., Masszi T., Lauri B., Levin M.D., Bosi A. (2018). Daratumumab plus bortezomib and dexamethasone versus bortezomib and dexamethasone in relapsed or refractory multiple myeloma: Updated analysis of CASTOR. Haematologica.

[94] Durig J., Naschar M., Schmucker U., Renzing-Kohler K., Holter T., Huttmann A., Duhrsen U. (2002). CD38 expression is an important prognostic marker in chronic lymphocytic leukaemia. Leukemia.

[95] Kishimoto H., Hoshino S., Ohori M., Kontani K., Nishina H., Suzawa M., Kato S., Katada T. (1998). Molecular mechanism of human CD38 gene expression by retinoic acid. Identification of retinoic acid response element in the first intron. J. Biol. Chem..

[96] Ferrero E., Saccucci F., Malavasi F. (1999). The human CD38 gene: Polymorphism, CpG island, and linkage to the CD157 (BST-1) gene. Immunogenetics.

[97] Malavasi F., Deaglio S., Damle R., Cutrona G., Ferrarini M., Chiorazzi N. (2011). CD38 and chronic lymphocytic leukemia: A decade later. Blood.

[98] Prus E., Fibach E. (2003). Retinoic acid induction of CD38 antigen expression on normal and leukemic human myeloid cells: Relationship with cell differentiation. Leuk. Lymphoma.

[99] Drach J., McQueen T., Engel H., Andreeff M., Robertson K.A., Collins S.J., Malavasi F., Mehta K. (1994). Retinoic acid-induced expression of CD38 antigen in myeloid cells is mediated through retinoic acid receptor-alpha. Cancer Res..

[100] Nijhof I.S., Groen R.W., Lokhorst H.M., van Kessel B., Bloem A.C., van Velzen J., de Jong-Korlaar R., Yuan H., Noort W.A., Klein S.K. (2015). Upregulation of CD38 expression on multiple myeloma cells by all-trans retinoic acid improves the efficacy of daratumumab. Leukemia.

[101] Frerichs K.A., Minnema M.C., Levin M.D., Broijl A., Bos G.M.J., Kersten M.J., Mutis T., Verkleij C.P.M., Nijhof I.S., Maas-Bosman P.W.C. (2021). Efficacy and safety of daratumumab combined with all-trans retinoic acid in relapsed/refractory multiple myeloma. Blood Adv..

[102] Wang Z., Liu Z., Wu X., Chu S., Wang J., Yuan H., Roth M., Yuan Y.C., Bhatia R., Chen W. (2014). ATRA-induced cellular differentiation and CD38 expression inhibits acquisition of BCR-ABL mutations for CML acquired resistance. PLoS Genet..

[103] Garcia-Guerrero E., Gogishvili T., Danhof S., Schreder M., Pallaud C., Perez-Simon J.A., Einsele H., Hudecek M. (2017). Panobinostat induces CD38 upregulation and augments the antimyeloma efficacy of daratumumab. Blood.

[104] Maiso P., Carvajal-Vergara X., Ocio E.M., Lopez-Perez R., Mateo G., Gutierrez N., Atadja P., Pandiella A., San Miguel J.F. (2006). The histone deacetylase inhibitor LBH589 is a potent antimyeloma agent that overcomes drug resistance. Cancer Res..

[105] Sanchez E., Shen J., Steinberg J., Li M., Wang C., Bonavida B., Chen H., Li Z.W., Berenson J.R. (2011). The histone deacetylase inhibitor LBH589 enhances the anti-myeloma effects of chemotherapy in vitro and in vivo. Leuk. Res..

[106] Garcia-Guerrero E., Gotz R., Doose S., Sauer M., Rodriguez-Gil A., Nerreter T., Kortum K.M., Perez-Simon J.A., Einsele H., Hudecek M. (2021). Upregulation of CD38 expression on multiple myeloma cells by novel HDAC6 inhibitors is a class effect and augments the efficacy of daratumumab. Leukemia.

[107] Wang H.F., Ning F., Liu Z.C., Wu L., Li Z.Q., Qi Y.F., Zhang G., Wang H.S., Cai S.H., Du J. (2017). Histone deacetylase inhibitors deplete myeloid-derived suppressor cells induced by 4T1 mammary tumors in vivo and in vitro. Cancer Immunol. Immunother..

[108] Ogiya D., Liu J., Ohguchi H., Kurata K., Samur M.K., Tai Y.T., Adamia S., Ando K., Hideshima T., Anderson K.C. (2020). The JAK-STAT pathway regulates CD38 on myeloma cells in the bone marrow microenvironment: Therapeutic implications. Blood.

[109] Endell J., Boxhammer R., Wurzenberger C., Ness D., Steidl S. (2012). The Activity of MOR202, a Fully Human Anti-CD38 Antibody, Is Complemented by ADCP and Is Synergistically Enhanced by Lenalidomide In Vitro and In Vivo. Blood.

[110] Endell J., Boxhammer R., Steidl S. (2014). Synergistic in Vitro Activity of MOR202, a Human CD38 Antibody, in Combination with Pomalidomide. Blood.

[111] Boxhammer R., Steidl S., Endell J. (2015). Effect of IMiD compounds on CD38 expression on multiple myeloma cells: MOR202, a human CD38 antibody in combination with pomalidomide. J. Clin. Oncol..

[112] Fedele P.L., Willis S.N., Liao Y., Low M.S., Rautela J., Segal D.H., Gong J.N., Huntington N.D., Shi W., Huang D.C.S. (2018). IMiDs prime myeloma cells for daratumumab-mediated cytotoxicity through loss of Ikaros and Aiolos. Blood.

[113] Peng Y., Croce C.M. (2016). The role of MicroRNAs in human cancer. Signal Transduct. Target. Ther..

[114] Hu Y., Liu H., Fang C., Li C., Xhyliu F., Dysert H., Bodo J., Habermehl G., Russell B.E., Li W. (2020). Targeting of CD38 by the Tumor Suppressor miR-26a Serves as a Novel Potential Therapeutic Agent in Multiple Myeloma. Cancer Res..

[115] Jude J.A., Dileepan M., Subramanian S., Solway J., Panettieri R.A., Walseth T.F., Kannan M.S. (2012). miR-140-3p regulation of TNF-alpha-induced CD38 expression in human airway smooth muscle cells. Am. J. Physiol. Lung Cell Mol. Physiol..

[116] Krejcik J., Frerichs K.A., Nijhof I.S., van Kessel B., van Velzen J.F., Bloem A.C., Broekmans M.E.C., Zweegman S., van Meerloo J., Musters R.J.P. (2017). Monocytes and Granulocytes Reduce CD38 Expression Levels on Myeloma Cells in Patients Treated with Daratumumab. Clin. Cancer Res..

[117] Nijhof I.S., Casneuf T., van Velzen J., van Kessel B., Axel A.E., Syed K., Groen R.W., van Duin M., Sonneveld P., Minnema M.C. (2016). CD38 expression and complement inhibitors affect response and resistance to daratumumab therapy in myeloma. Blood.

[118] van de Donk N., Usmani S.Z. (2018). CD38 Antibodies in Multiple Myeloma: Mechanisms of Action and Modes of Resistance. Front. Immunol..

[119] Chillemi A., Zaccarello G., Quarona V., Ferracin M., Ghimenti C., Massaia M., Horenstein A.L., Malavasi F. (2013). Anti-CD38 antibody therapy: Windows of opportunity yielded by the functional characteristics of the target molecule. Mol. Med..

[120] Funaro A., Reinis M., Trubiani O., Santi S., Di Primio R., Malavasi F. (1998). CD38 functions are regulated through an internalization step. J. Immunol..

[121] Taylor R.P., Lindorfer M.A. (2015). Fcgamma-receptor-mediated trogocytosis impacts mAb-based therapies: Historical precedence and recent developments. Blood.

[122] Chillemi A., Quarona V., Zito A., Morandi F., Marimpietri D., Cuccioloni M., Robert O.J., Mark C.S., Bolzoni M., Toscani D. (2015). Generation and Characterization of Microvesicles after Daratumumab Interaction with Myeloma Cells. Blood.

[123] Malavasi F., Faini A.C., Morandi F., Castella B., Incarnato D., Oliviero S., Horenstein A.L., Massaia M., van de Donk N., Richardson P.G. (2021). Molecular dynamics of targeting CD38 in multiple myeloma. Br. J. Haematol..

[124] Mustafa N., Azaman M.I., Chng W.J. (2021). Daratumumab Resistant Natural Killer/T-Cell Lymphoma Exhibit an Addiction to the Exosome Biogenesis Pathway for Survival. Blood.

[125] Davies F.E., Raje N., Hideshima T., Lentzsch S., Young G., Tai Y.T., Lin B., Podar K., Gupta D., Chauhan D. (2001). Thalidomide and immunomodulatory derivatives augment natural killer cell cytotoxicity in multiple myeloma. Blood.

[126] van der Veer M.S., de Weers M., van Kessel B., Bakker J.M., Wittebol S., Parren P.W., Lokhorst H.M., Mutis T. (2011). Towards effective immunotherapy of myeloma: Enhanced elimination of myeloma cells by combination of lenalidomide with the human CD38 monoclonal antibody daratumumab. Haematologica.

[127] Kortum K.M., Zhu Y.X., Shi C.X., Jedlowski P., Stewart A.K. (2015). Cereblon binding molecules in multiple myeloma. Blood Rev..

[128] Gavriatopoulou M., Kastritis E., Ntanasis-Stathopoulos I., Fotiou D., Roussou M., Migkou M., Ziogas D.C., Kanellias N., Terpos E., Dimopoulos M.A. (2018). The addition of IMiDs for patients with daratumumab-refractory multiple myeloma can overcome refractoriness to both agents. Blood.

[129] Verkleij C.P.M., Jhatakia A., Broekmans M.E.C., Frerichs K.A., Zweegman S., Mutis T., Bezman N.A., van de Donk N. (2020). Preclinical Rationale for Targeting the PD-1/PD-L1 Axis in Combination with a CD38 Antibody in Multiple Myeloma and Other CD38-Positive Malignancies. Cancers.

[130] Wang X., Yu X., Li W., Neeli P., Liu M., Li L., Zhang M., Fang X., Young K.H., Li Y. (2022). Expanding anti-CD38 immunotherapy for lymphoid malignancies. J. Exp. Clin. Cancer Res..

[131] Teoh P.J., Chng W.J. (2021). CAR T-cell therapy in multiple myeloma: More room for improvement. Blood Cancer J..

[132] Feng Y., Liu X., Li X., Zhou Y., Song Z., Zhang J., Shi B., Wang J. (2021). Novel BCMA-OR-CD38 tandem-dual chimeric antigen receptor T cells robustly control multiple myeloma. Oncoimmunology.

[133] Cui Q., Qian C., Xu N., Kang L., Dai H., Cui W., Song B., Yin J., Li Z., Zhu X. (2021). CD38-directed CAR-T cell therapy: A novel immunotherapy strategy for relapsed acute myeloid leukemia after allogeneic hematopoietic stem cell transplantation. J. Hematol. Oncol..

[134] Fayon M., Martinez-Cingolani C., Abecassis A., Roders N., Nelson E., Choisy C., Talbot A., Bensussan A., Fermand J.P., Arnulf B. (2021). Bi38-3 is a novel CD38/CD3 bispecific T-cell engager with low toxicity for the treatment of multiple myeloma. Haematologica.

[135] Saltarella I., Desantis V., Melaccio A., Solimando A.G., Lamanuzzi A., Ria R., Storlazzi C.T., Mariggio M.A., Vacca A., Frassanito M.A. (2020). Mechanisms of Resistance to Anti-CD38 Daratumumab in Multiple Myeloma. Cells.

[136] Franssen L.E., Stege C.A.M., Zweegman S., van de Donk N., Nijhof I.S. (2020). Resistance Mechanisms Towards CD38-Directed Antibody Therapy in Multiple Myeloma. J. Clin. Med..

[137] Wang Y., Zhang Y., Hughes T., Zhang J., Caligiuri M.A., Benson D.M., Yu J. (2018). Fratricide of NK Cells in Daratumumab Therapy for Multiple Myeloma Overcome by Ex Vivo-Expanded Autologous NK Cells. Clin. Cancer Res..

[138] Naeimi Kararoudi M., Nagai Y., Elmas E., de Souza Fernandes Pereira M., Ali S.A., Imus P.H., Wethington D., Borrello I.M., Lee D.A., Ghiaur G. (2020). CD38 deletion of human primary NK cells eliminates daratumumab-induced fratricide and boosts their effector activity. Blood.

[139] You T., Hu W., Ge X., Shen J., Qin X. (2011). Application of a novel inhibitor of human CD59 for the enhancement of complement-dependent cytolysis on cancer cells. Cell Mol. Immunol..

[140] Macor P., Secco E., Mezzaroba N., Zorzet S., Durigutto P., Gaiotto T., De Maso L., Biffi S., Garrovo C., Capolla S. (2015). Bispecific antibodies targeting tumor-associated antigens and neutralizing complement regulators increase the efficacy of antibody-based immunotherapy in mice. Leukemia.

